# Correction: Abdel-Raheem et al. Double-Fermented Soybean Meal Totally Replaces Soybean Meal in Broiler Rations with Favorable Impact on Performance, Digestibility, Amino Acids Transporters and Meat Nutritional Value. *Animals* 2023, *13*, 1030

**DOI:** 10.3390/ani16213421

**Published:** 2026-10-29

**Authors:** Sherief M. Abdel-Raheem, El Said Yehia Mohammed, Rania Elsaid Mahmoud, Mahmoud Fathy El Gamal, Hend S. Nada, Waleed Rizk El-Ghareeb, Mohamed Marzok, Ahmed M. A. Meligy, Mohamad Abdulmohsen, Hesham Ismail, Doaa Ibrahim, Asmaa T. Y. Kishawy

**Affiliations:** 1 Department of Public Health, College of Veterinary Medicine, King Faisal University, Al-Ahsa, Hofuf 31982, Saudi Arabia; 2 Department of Animal Nutrition and Clinical Nutrition, Faculty of Veterinary Medicine, Assiut University, Assiut 71526, Egypt; 3 Department of Nutrition and Clinical Nutrition, Faculty of Veterinary Medicine, Zagazig University, Zagazig 44519, Egypt; 4 Department of Microbiology, Faculty of Veterinary Medicine, Zagazig University, Zagazig 44519, Egypt; 5 Food Control Department, Faculty of Veterinary Medicine, Zagazig University, Zagazig 44519, Egypt; 6 Department of Clinical Sciences, College of Veterinary Medicine, King Faisal University, Al-Ahsa, Hofuf 31982, Saudi Arabia; 7 Department of Surgery, Faculty of Veterinary Medicine, KafrelSheikh University, KafrelSheikh 33511, Egypt; 8 Department of Physiology, Agricultural Research Center (ARC), Giza 13611, Egypt; 9 Department of Animal Behavior, Faculty of Veterinary Medicine, Suez Canal University, Ismailia 41522, Egypt; 10 Food Hygiene Department, Faculty of Veterinary Medicine, Assiut University, Assiut 71526, Egypt

## Text Correction

There was an error in the original publication [1]. Due to the authors’ oversight, there was a typographical error in the percentage values of free amino acids, fiber, lipid, and trypsin inhibitor in the Abstract. A correction has been made to the Abstract as follows:

Inclusion of microbial fermented soybean meal in broiler feed has induced advantageous outcomes for their performance and gastrointestinal health by exhibiting probiotic effects. In this study, soybean meal (SBM) was subjected to double-stage microbial fermentation utilizing functional metabolites of fungi and bacteria. In the broiler diet, DFSBM replaced SBM at 0%, 25%, 50%, and 100%. DFSBM was reported to have higher protein content and total essential, nonessential, and free amino acids (increased by 3.67%, 12.81%, 10.10%, and 6.53)-fold, respectively, compared to SBM. Notably, phytase activity and lactic acid bacteria increased, while fiber, lipid, and trypsin inhibitor contents decreased by 13.82%, 38.31%, and 73.50%, respectively, in a diet containing 100% DFSBM compared to SBM. Improved growth performance and apparent nutrient digestibility, including phosphorus and calcium, and pancreatic digestive enzyme activities were observed in groups fed higher DFSBM levels. In addition, higher inclusion levels of DFSBM increased blood immune response (IgG, IgM, nitric oxide, and lysozyme levels) and liver antioxidant status. Jejunal amino acids- and peptide-transporter-encoding genes (LAT1, CAT-1, CAT-2, PepT-1, and PepT-2) were upregulated with increasing levels of DFSBM in the ration. Breast muscle crude protein, calcium, and phosphorus retention were increased, especially at higher inclusion levels of DFSBM. Coliform bacteria load was significantly reduced, while lactic acid bacteria count in broiler intestines was increased with higher dietary levels of DFSBM. In conclusion, replacement of SBM with DFSBM positively impacted broiler chicken feed utilization and boosted chickens’ amino acid transportation, in addition to improving the nutritional value of their breast meat.

The authors state that the scientific conclusions are unaffected. This correction was approved by the Academic Editor. The original publication has also been updated.
